# RegnANN: Reverse Engineering Gene Networks Using Artificial Neural Networks

**DOI:** 10.1371/journal.pone.0028646

**Published:** 2011-12-28

**Authors:** Marco Grimaldi, Roberto Visintainer, Giuseppe Jurman

**Affiliations:** Fondazione Bruno Kessler, Trento, Italy; University of Turin, Italy

## Abstract

RegnANN is a novel method for reverse engineering gene networks based on an ensemble of multilayer perceptrons. The algorithm builds a regressor for each gene in the network, estimating its *neighborhood* independently. The overall network is obtained by joining all the neighborhoods. RegnANN makes no assumptions about the nature of the relationships between the variables, potentially capturing high-order and non linear dependencies between expression patterns. The evaluation focuses on synthetic data mimicking plausible submodules of larger networks and on biological data consisting of submodules of *Escherichia coli*. We consider Barabasi and Erdös-Rényi topologies together with two methods for data generation. We verify the effect of factors such as network size and amount of data to the accuracy of the inference algorithm. The accuracy scores obtained with RegnANN is methodically compared with the performance of three reference algorithms: ARACNE, CLR and KELLER. Our evaluation indicates that RegnANN compares favorably with the inference methods tested. The robustness of RegnANN, its ability to discover second order correlations and the agreement between results obtained with this new methods on both synthetic and biological data are promising and they stimulate its application to a wider range of problems.

## Introduction

The task of gene regulatory network (GRN) inference is a daunting task not only in terms of devising an effective algorithm, but also in terms of quantitatively interpreting the obtained results [1]. Only recently efforts have been carried out towards an objective comparison of network inference methods also highlighting occurring limitations, e.g.: [2]–[5]. As an example, the Dialogue for Reverse Engineering Assessments and Methods (DREAM) challenge is one of the prominent efforts that aims at evaluating the success of GRN inference algorithms on synthetic benchmarks data sets.

Early network inference models were based on the analysis of the correlation coefficients [6], [7] between expression patterns of all pairs of genes to infer *co-expression* networks. On the basis that correlation coefficients fail to capture more complex statistical dependencies among expression patterns (e.g. non-linearity), more recently general methods based on measures of dependency such as mutual information have been proposed [7]. Of this class of algorithms, ARACNE [8] and CLR [9] have been adopted to address a wide range of network deconvolution problems - from transcriptional [8] to metabolic networks [10] - and they are often used as reference benchmark algorithms (e.g.: [4], [7], [11], [12]). Generally, the inference methods proposed are of very different nature, ranging from deterministic (systems of differential equations [13] and Gröbner bases [14]) to stochastic approaches, e.g.: Boolean [15] or Bayesian [16] algorithms. Such approaches may also start from different types of gene expression data: time-course or steady states. Furthermore, the detail and the complexity of the considered network can also vary: links may carry information about the direction of the relation (directed graph) and a weight may be associated to the strength of each link (weighted graph) [17], [18]. Generally, the reconstruction accuracy is far from being optimal due to drawbacks related to both the methods and the available data [19].

One of the aspects that makes network inference a daunting task is its intrinsic underdetermination [2] related to the size of the search space. It is often the case that the expression profiles of thousands of genes (e.g. approximately 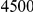 in the case of *Escherichia coli*), controlled by hundreds of regulators (e.g. approximately 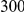 known transcription factors in *Escherichia coli*), are recorded for a limited amount of experimental conditions - about 450 for the last publicly available *Escherichia coli* gene expression data set. Thus, considering also possible combinatorial regulations and feedback loops, the number of possible solutions of the inference problem becomes prohibitively large compared to the available experimental measurements at hand.

In this work we introduce a novel inference method called Reverse Engineering Gene Networks with Artificial Neural Networks (RegnANN). This inference algorithm, trained using steady state data as provided by microarray data, builds a multi-variable regressor (one to many) for each gene in the network. The algorithm is based on an ensemble of Multilayer Perceptrons (MLPs) trained using steady state data. RegnANN estimates the *neighborhood* of each gene (the correlations among one gene and all the others) independently and then it joins these neighborhoods to form the overall network. RegnANN performance is compared with those of top-scoring methods such as KELLER [20], ARACNE [8] and CLR [9]. To improve the general efficiency of RegnANN we implement the algorithm using the GPGPU programming paradigm [21]. The main feature of the novel method presented is that it makes no assumptions about the nature of the relationships between the variables, potentially capturing high-order and non linear dependencies between expression patterns. On the other hand, RegnANN differs greatly from other published methods based on ANN or simple binary perceptron (e.g.: [22]–[26]): it is a multi-variable regressor (one input variable, many output variables) trained using steady states data for determining gene interactions.

In evaluating the performance of the four different network inference methods, first we settle in a controlled situation with synthetic data and then we focus on a biological setup by analyzing transcriptional subnetworks of *Escherichia coli*.

The general performance of the network inference task is evaluated in terms of Matthews Correlation Coefficient - MCC [27]. MCC is becoming the accuracy measure of choice in many application fields of machine learning and bioinformatics: it is one of the best methods for summarizing into a single value the confusion matrix of a binary classification task, recently adopted also for network topology comparison [28].

The experimental evaluation firstly verifies RegnANN ability of inferring direct and indirect interactions among genes and possible cooperative interaction between putative regulators on a set of toy experiments. Considering only the underlying topology (e.g.: undirected unweighted graph), we then focus on synthetic data mimicking plausible submodules of larger networks generated according to both Barabasi [29] and Erdös-Rényi [30] models. In doing so, we focus on a scenario of reduced search space/extended amount of independent information [2]. To tackle various aspects of the problem of network inference, we analyze the effect of, e.g.: increasing the amount of available data while varying the topology of the network, the number of nodes in the network, the data synthesis method and the inference algorithm applied.

We finally demonstrate our approach on a biological data set consisting of a selected number of subnetworks of *Escherichia coli* including a number of genes ranging from 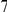 to 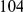. The expression data consists of 445 microarray expression profiles collected under different experimental conditions.

## Results

In order to present coherently the results obtained on synthetic data, we start firstly with an evaluation of RegnANN ability of inferring direct and indirect interactions among genes and possible cooperative interaction between putative regulators (e.g.: transcription factors). This is done on four toy examples considering interaction among four genes.

The second phase of our analysis focuses on the effect of varying the amount of available data in the task of network inference while considering a fixed threshold for the binarization of the inferred adjacency matrix. The performance in terms of MCC obtained with RegnANN is systematically compared with the ones obtained by KELLER. The accuracy of each inference method is firstly evaluated on synthetic data by varying the topology of the network, the amount of data available and the method adopted to synthesize the data. Once the topology of the network is (randomly) selected, the desired amount of data is synthesized according to the generation method of choice, the network inference methods are applied and the MCC score calculated. We consider discrete (in {0, 1}) symmetric adjacency matrices for fair comparison between the two methods as KELLER does not infer coupling direction, nor the strength of the interaction. To account for intrinsic instability of each inference method, data generation and network inference are repeated 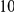 times for each given network topology and the MCC score is estimated as the mean of the 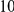 independent runs. The error of the measurement is expressed as twice the standard deviation. In order to generalize from the selected network topology, the entire procedure is repeated 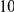 times and the final accuracy score (MCC) is calculated as the mean accuracy for each run. Similarly, the error of the accuracy score is estimated as the mean of the error for each run. Our analysis explores the effect of varying the mean degree of the nodes in the case of Erdös-Rényi networks and the exponent of the power-law for Barabasi networks in network inference. We also test the effect of different data normalization procedures on the accuracy of the two network induction methods considered.

The third phase of the experimental evaluation on synthetic data compares the performance of RegnANN, ARACNE and CLR in terms of AUC (the area under the curve). The curve is constructed by varying the value of the binarization threshold between 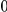 and the maximum score obtained by the given inference method on the task at hand - in the case of RegnANN the maximum value is bounded to 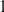, while it is not the case for ARACNE and CLR. As in the previous phase, this is done while varying the topology of the network, the number of nodes and verifying the effect of different mean degrees and power-law coefficients. Also in this phase we consider discrete symmetric adjacency matrices for fair comparison between the methods: ARACNE and CLR do not infer coupling direction. For homogeneity, we introduce the MR (MCC-Recall) curve which express the MCC value for the corresponding Recall value. Although the Precision-Recall (PR) curve is a well known tool in assessing the performance of an induction algorithm, the MR is an equivalent measure that has a straightforward interpretation. The major difference between the two curves is that an induction algorithm scoring a 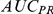 value of 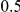 has performance substantially equivalent to chance, the same induction system would score an 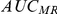 value of 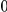. In perfect analogy with the previous phase, data generation and network inference are repeated 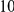 times for each given network topology and the AUC score is estimated as the mean of the 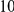 independent runs. The error of the measurement is expressed as twice the standard deviation.

Finally, we compare the results obtained on a selection of Escherichia coli gene subnetworks for the four inference algorithms. We will first start considering a fixed threshold for the binarization of the inferred adjacency matrix. Secondly, we briefly analyze the problem of optimal threshold selection for the binarization of the inferred adjacency matrix in the hypothesis of the presence of gold standard data, which is not available in most realistic biological applications. As for the two phases before, we consider discrete symmetric adjacency matrices.

### Toy Examples

In this section we present four different toy experiments to illustrate how RegnANN is capable of inferring direct and indirect interactions among genes and cooperative interaction between putative regulators (e.g.: transcription factors).

### Single Interaction

**Figure 1:**
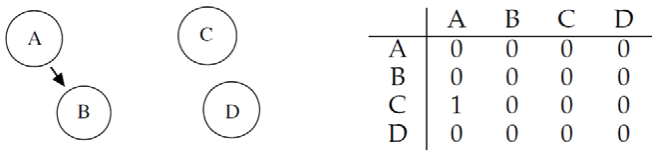
Left: gene 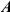 regulates gene 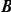, while 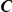 and 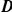 do not interact with any other. Right: corresponding adjacency matrix.

Let us consider four different genes 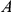, 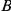, 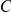 and 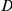, which interact according to Figure 1: gene 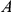 regulates 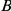, while 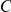 and 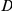 do not interact with anyone. We assume that 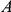 is a regulator that can be in an active or inactive state. If it is in an active state, it has the effect of a linear regulation on B. If it is in an inactive state, B is driven by noise. We generate 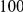 expression profiles for the four genes as follows: value of 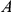 is 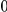 with probability 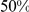 (A is inactive), and uniformly distributed in 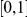 with probability 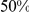. The value of gene 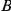 is uniformly distributed in the interval 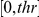 if 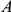 is inactive (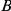 values are driven by noise), otherwise 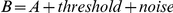 (in the case the values of 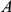 are different from 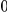). The term 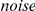 is a value uniformly distributed in the interval 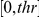. Expression values for genes 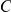 and 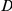 are uniformly distributed in the interval 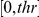 - C and D are entirely driven by noise. The value for the threshold 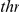 is set arbitrarily to 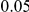. Before inference, gene expression profiles are linearly rescaled in 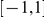.

**Figure 2:**
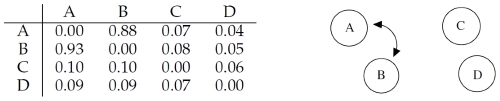
Left: mean correlation values inferred with RegnANN. Right: inferred interaction among the genes for correlation greater than 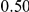.

The correlation matrix shown in Figure 2 indicates that RegnANN is able to capture the correlation among genes 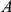 and 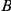: for interaction 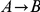, RegnANN calculates a correlation of 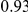 (and a correlation of 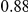 for the opposite direction: 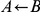). On the other hand no correlation (equal or less than 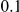) is recorded among 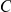, 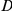 and the other genes. It is interesting to note that, if we select the direction of interaction by identifying the highest correlation value, RegnANN successfully identifies the regulation as in Figure 1.

### Cooperative Interaction

**Figure 3:**
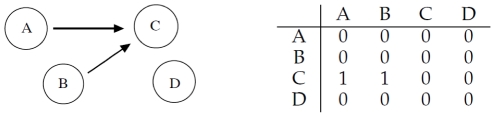
Left: gene 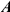 and 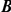 cooperatively regulate gene 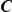, while 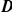 does not interact with the other three. Right: corresponding adjacency matrix.

Let us consider four different genes 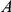, 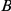, 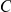 and 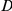, which interact according to Figure 3: gene 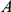 and gene 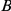 cooperatively regulate gene 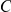, while 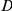 does not interact with the other three.

**Figure 4:**
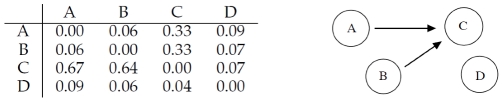
Left: mean correlation values inferred with RegnANN. Right: inferred interaction among the genes for correlation greater than 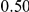.

We synthesize expression profiles very similarly to the SLC method: we start considering a set of 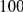 *seed* expressions with values uniformly distributed in 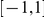 for each gene 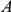, 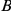, 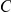, 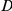. In order to simulate the presence of an activation threshold, we calculate the expression value for the genes as follows:
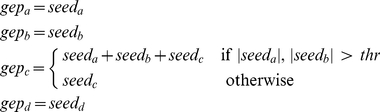
where 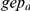, 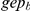, 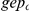 and 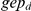 are the gene expression profiles for gene 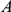, 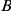, 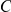 and 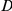 respectively, while 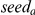, 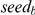, 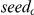 and 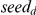 are the corresponding seed values. The value for the threshold 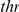 is set arbitrarily to 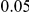. After generation the profiles are rescaled linearly in 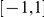. To account for intrinsic instability of the inference method, data generation and network inference are repeated 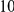 times and the adjacency matrix accumulated. Figure 4 shows the mean correlation values inferred by RegnANN (left) and the obtained interaction among genes for correlation greater than 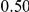 (right).

The correlation matrix shown in Figure 4 indicates that RegnANN is able to capture the correlation among genes 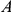, 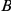 and 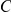: for interaction 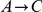, RegnANN calculates a correlation of 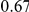 (and a correlation of 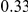 for the opposite direction: 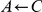). Similar correlation values are recoded for 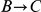 and 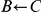. It is interesting to note that no correlation (less than 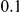) is recorded among 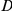 and the other genes.

### Multiple Interaction

**Figure 5:**
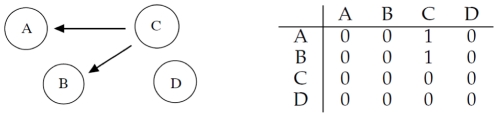
Left: gene 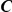 regulates gene 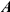 and gene 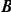, while 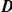 does not interact with the other three. Right: corresponding adjacency matrix.

Let us consider again the four different genes 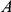, 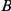, 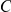 and 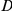 interacting according to Figure 5: gene 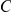 regulates gene 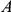 and gene 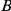, while 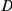 does not interact with the other three.

**Figure 6:**
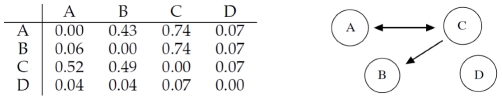
Left: mean correlation values inferred with RegnANN. Right: inferred interaction among the genes for correlation greater than 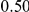.

As in the previous case, we generate the gene expression profiles considering a set of 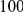 *seed* expressions with values uniformly distributed in [−1, 1] for each gene 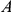, 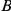, 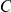, 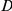. We calculate the expression value for the genes as follows:
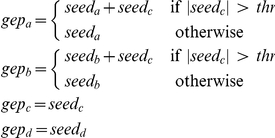
where 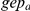, 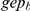, 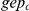 and 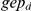 are the gene expression profiles for gene 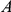, 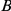, 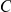 and 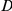 respectively, while 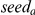, 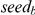, 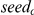 and 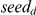 are the corresponding seed values. The value for the threshold 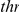 is set arbitrarily to 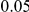. Figure 6 shows the mean correlation values for the 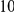 data generation/inference iterations (left) and the obtained interaction among genes for correlation greater than 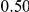 (right).

The correlation matrix in Figure 6 indicates that both 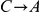 and 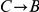 interactions are discovered (a correlation of about 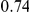 in both cases), while a weak interaction 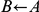 is recorded (correlation 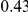). Other two weak interactions are also reported: 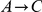 (correlation 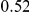) and 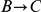 (correlation 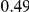). Strictly considering the adjacency matrix in Figure 5 and a discretization threshold of 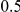, one false link is recorded (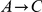) in the inferred interaction matrix.

### Indirect Interaction

**Figure 7:**
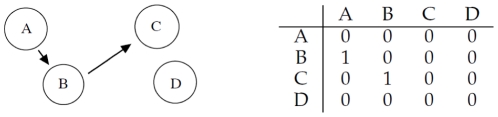
Left: gene 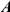 regulates gene 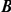 and gene 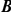 regulates gene 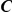, while 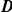 does not interact with the other three. Right: corresponding adjacency matrix.

As a final example, let us consider the four different genes 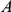, 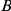, 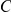 and 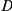 interacting according to Figure 7: gene 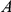 regulates gene 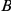 and gene 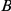 regulates gene 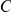, while 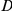 does not interact with the other three genes.

**Figure 8:**
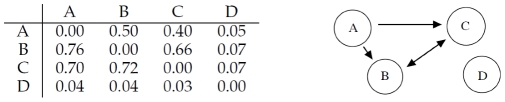
Left: mean correlation values inferred with RegnANN. Right: inferred interaction among the genes for correlation greater than 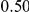.

We generate the gene expression profiles considering a set of 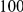 *seed* expressions with values uniformly distributed in 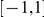 for each gene 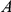, 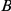, 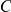, 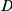. We calculate the expression values for the genes as follows:
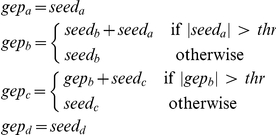



Figure 8 shows the mean correlation values for the 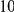 data generation/inference iterations (left) and the obtained interaction among genes for correlation strictly greater than 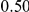 (right).

The correlation matrix in Figure 8 indicates that interaction 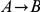 and interaction 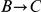 are correctly identified with the highest correlation values (correlation 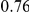 and correlation 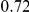 respectively). A third strong interaction is also indicated by RegnANN: 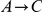. The latter is in fact a second order interaction between the the two genes. In Table 1, Table 2 and Table 3 we report for comparison the inferred mutual information matrix inferred by ARACNE, the mean scores obtained applying CLR and the mean adjacency matrix inferred by KELLER on the same exercise.

**Table 1:** Mean mutual information values over 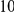 runs obtained applying ARACNE for inferring indirect interactions.

	A	B	C	D
A	0.00	0.48	0.00	0.00
B	0.48	0.00	0.63	0.00
C	0.00	0.63	0.00	0.00
D	0.00	0.00	0.00	0.00

**Table 2:** Mean scores over 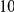 runs obtained applying CLR for inferring indirect interactions.

	A	B	C	D
A	0.00	1.87	0.38	1.01
B	1.87	0.00	2.73	0.90
C	0.38	2.73	0.00	0.58
D	1.01	0.90	0.58	0.00

**Table 3:** Mean adjacency matrix obtained applying KELLER for inferring indirect interactions.

	A	B	C	D
A	0.00	1.00	0.30	0.00
B	1.00	0.00	1.00	0.00
C	0.30	1.00	0.00	0.00
D	0.00	0.00	0.00	0.00

Value 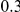 indicates that the link has been detected 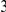 times in 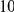 runs.

Strictly considering the adjacency matrix in Figure 7, RegnANN finds 2 false links (Figure 8): 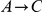 and 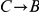 (if we include correlation value 0.50, the interaction 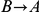 would be a third false positive).

In order to eliminate the ambiguity in the determination of the direction of the interaction among genes, in the following we will consider symmetric adjacency matrices as input for data generation and as the output of the network inference task. As a second possible solution, in the case of RegnANN we could have selected the direction of interaction by identifying the highest correlation value. On the other hand, this choice would have resulted in an unfair comparison with (i.e.) KELLER: the latter discards any information about the direction of the interaction. However, it is important to consider that an exhaustive analysis of the direction of the coupling would require a dedicated procedure to account for the variability of the regression.

### Synthetic Networks

In this section we analyze the performance of RegnANN in inferring network topology by applying a fixed discretization threshold of 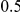 on the correlation values obtained. These results are systematically compared with the ones obtained by the KELLER algorithm on the same set of tasks. Data generation and network inference are repeated 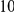 times for each given network topology and the MCC score is estimated as the mean of the 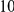 independent runs. The error of the measurement is expressed as twice the standard deviation.

### Effect of sample availability

**Figure 9:**
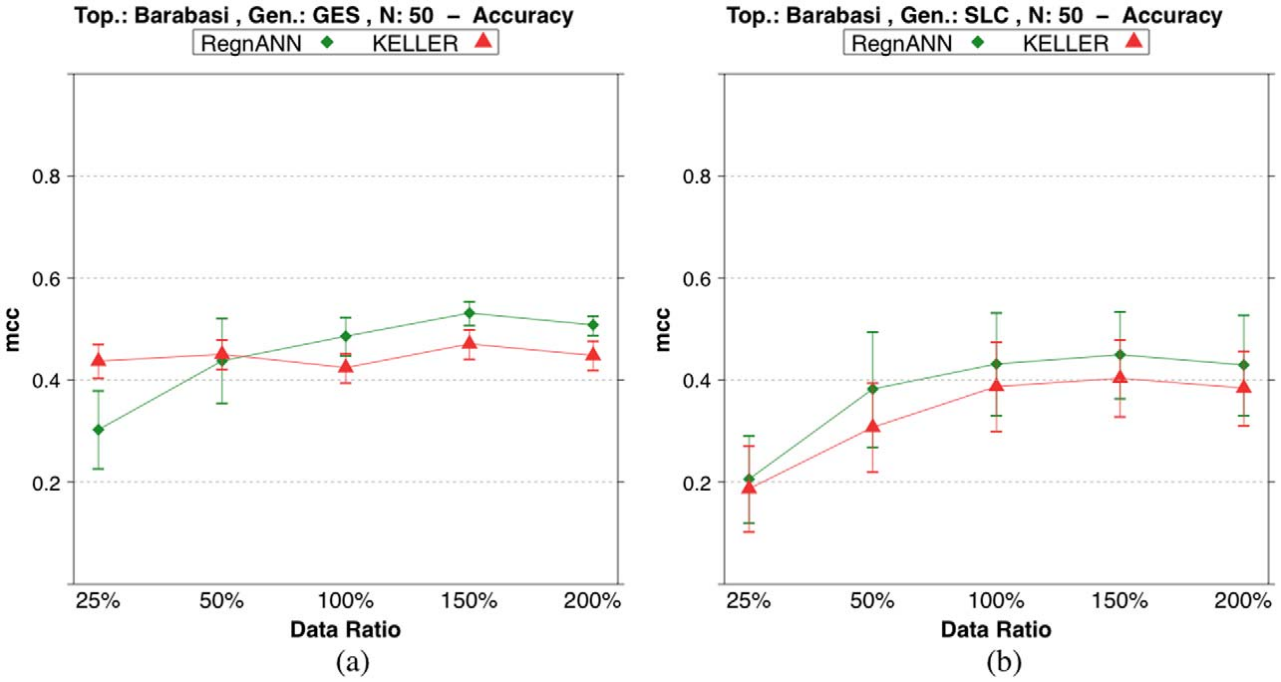
Accuracy (MCC) scores of RegnANN and KELLER on synthetic Barabasi networks with 50 nodes. (a) Results obtained with GES data generation. (b) Results obtained with SLC data generation.

Figure 9 shows accuracy (MCC) scores on synthetic Barabasi networks with 50 nodes while varying data synthesis methodology and *data ratio*: the ratio of the number of expression profiles to the number of nodes. We rescale linearly the synthetic gene expression values in 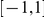. Moreover, we generate Barabasi networks with power-law equal to 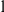 and Erdös-Rényi networks with mean degree equal to 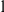. Figure 9 indicates that increasing the available data is beneficial to the performance of all the inference methods tested irrespective of the data synthesis method applied. RegnANN-SLC - Figure 9(b) - shows MCC scores ranging between 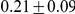 and 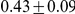 for data ratio 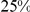 and data ratio 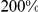 respectively. For the same values of data ratio, KELLER-SLC scores 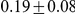 and 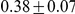. Similar behavior is recorded for GES data synthesis - Figure 9(a).

**Figure 10:**
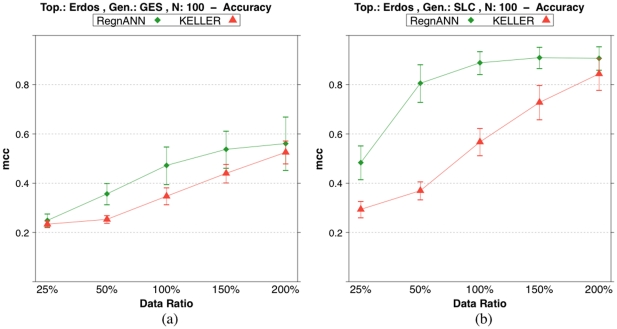
Accuracy (MCC) scores of RegnANN and KELLER on synthetic Erdös-Rényi networks with 100 nodes. (a) Results obtained with GES data generation. (b) Results obtained with SLC data generation.

Figure 10 shows accuracy scores on synthetic Erdös-Rényi networks with 100 nodes while varying data synthesis methodology and data ratio. In the case of GES data synthesis, RegnANN scores 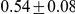 for data ratio 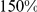. Similarly, KELLER scores 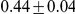 for data ratio 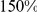. In the case of SLC and for data ratio 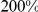, RegnANN scores 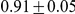, KELLER scores 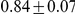.

**Figure 11:**
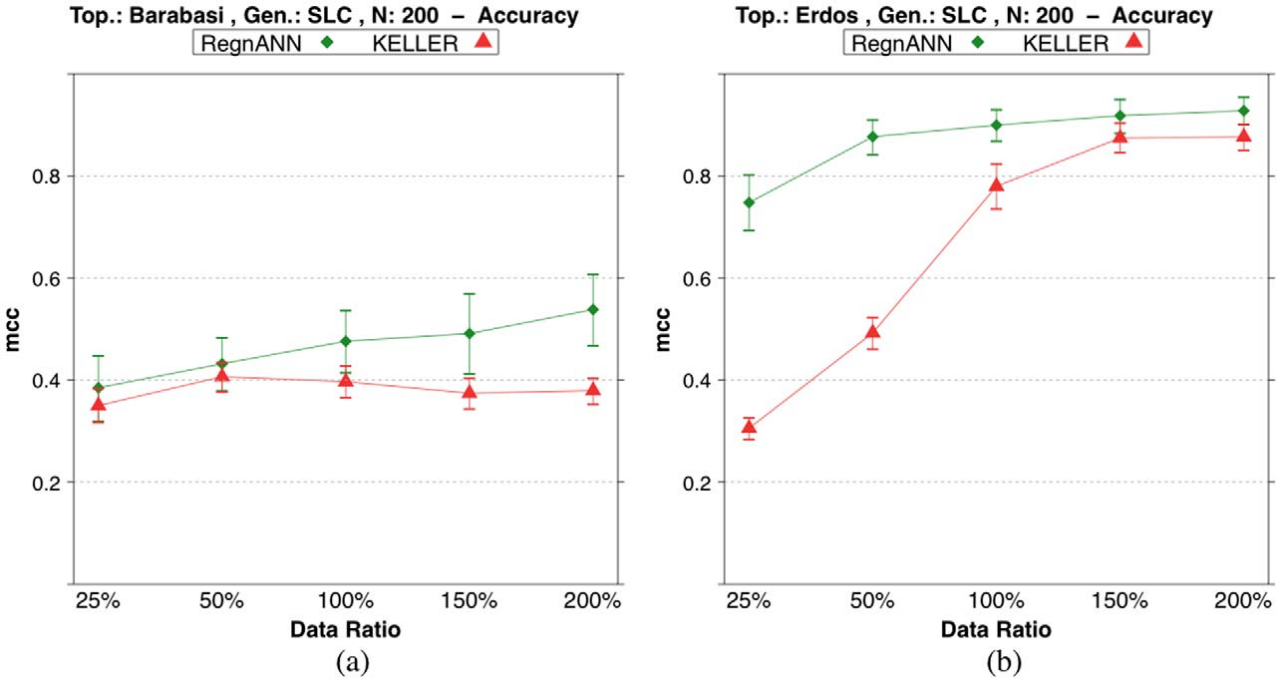
Accuracy (MCC) scores of RegnANN and KELLER on synthetic networks with 200 nodes, SLC data generation. (a) Results obtained for Barabasi topology. (b) Results obtained for Erdös-Rényi topology.

Figure 11 shows accuracy scores on synthetic Erdös-Rényi and Barabasi networks with 200 nodes while varying the data ratio. The figure shows very good performance of RegnANN on both Barabasi and Erdös-Rényi topology. The method we propose shows accuracy scores constantly above the MCC values obtained with the other method. It is interesting to note that the two different topologies have significative influence on the accuracies of the methods tested. Considering data ratio 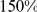, RegnANN scores 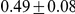 and 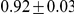 on Barabasi and Erdös-Rényi networks respectively.

**Figure 12:**
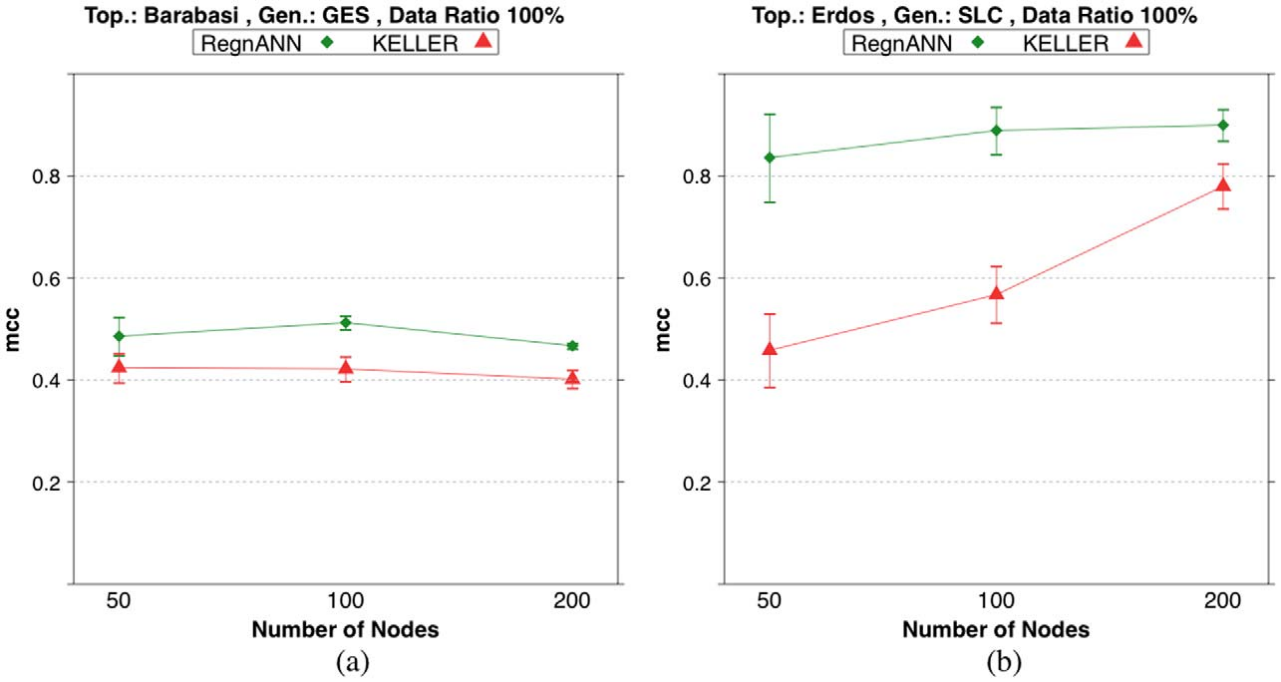
Accuracy (MCC) scores of RegnANN and KELLER on synthetic networks with Data Ratio 100% while varying number of nodes. (a) Results obtained for Barabasi topology, GES data generation. (b) Results obtained for Erdös-Rényi topology, SLC data generation.

Figure 12 summarizes the accuracy scores on synthetic Erdös-Rényi and Barabasi networks obtained with Data Ratio 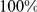 while varying number of nodes and data generation method.

### Effect of mean degree and power-law coefficient

In the following we show results obtained by varying the mean degree of the nodes between 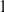 and 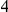 for Erdös-Rényi networks We also test the performance of the two inference algorithms by varying the exponent of the power-law in the range 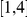 for Barabasi networks. Here we consider synthetic networks of 100 nodes, SLC data generation and expression linearly rescaled in 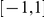.

**Figure 13:**
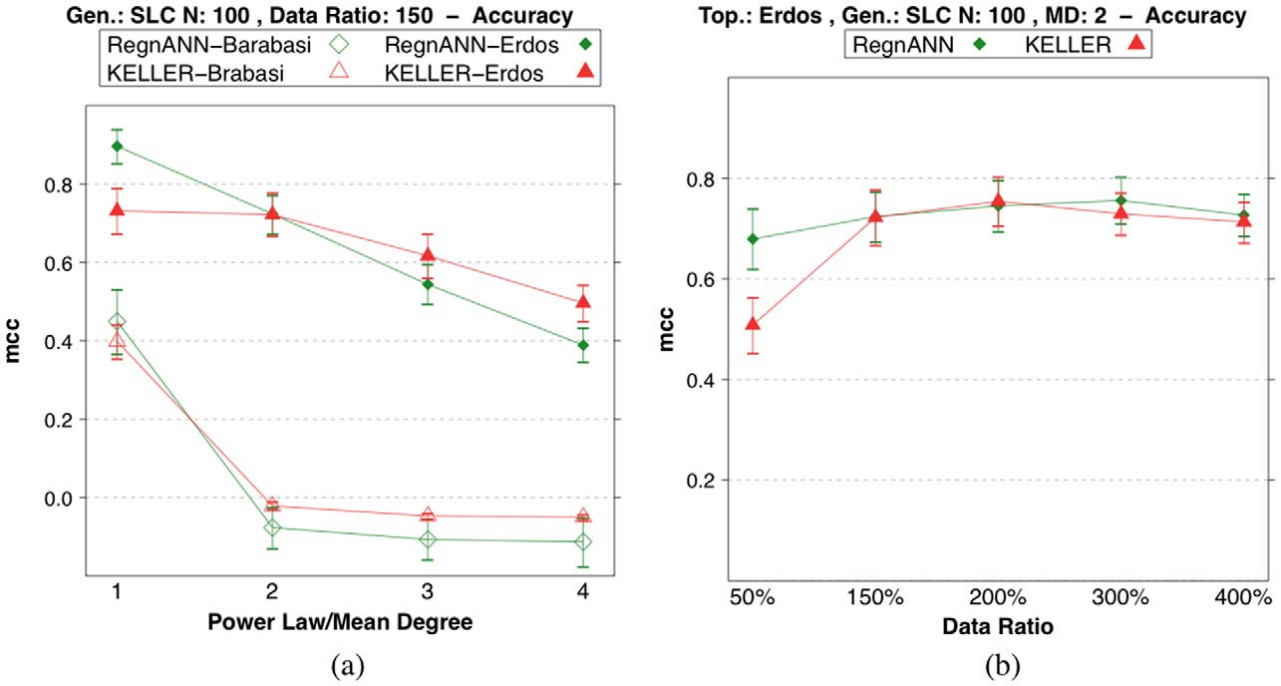
Accuracy (MCC) scores of RegnANN and KELLER on synthetic networks with 100 nodes and SLC data generation. (a) Results obtained on Barabasi networks varying the power-law coefficient and Erdös-Rényi topology while varying the mean degree of the network. (b) Results obtained for Erdös-Rényi (mean degree value equal to 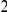) while varying the data ratio.

Figure 13(a) shows accuracy scores that decrease as the mean degree/power-law coefficient increases.

In the case of Erdös-Rényi topology with mean degree equal to 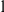, RegnANN scores 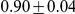; in the case of mean degree equal to 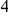, the same algorithm scores 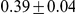. Also KELLER shows an accuracy curve for the Erdös-Rényi topology that decreases as the mean degree of the network increases, although this behavior is less marked.

In the case of Barabasi topology, the accuracy of both methods drops quickly to a value of 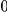 as the exponent of the power-law increases.

It is interesting to note that for both topologies, KELLER tends to outperform RegnANN for mean degree values bigger and equal to 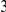, when we consider a data ratio of 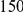.

Figure 13(b) shows the accuracy score of Erdös-Rényi networks with mean degree equal to 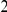. As in the case of Erdös-Rényi networks with mean degree equal to 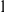 - Figure 10(b) - the performance of the two methods tends to increase as the amount of data available increases.

### Effect of data normalization

In microarray experiments, the analysis of the raw data is often hampered by a number of technical and statistical problems. The possible remedies usually lie in appropriate preprocessing steps, proper normalization of the data and application of statistical testing procedures in the derivation of differentially expressed genes [31]. Although many of the real-world issues in data preprocessing and normalization do not apply here, we are interested in verifying how discretization and rescaling - some of the most common (and possibly simple) steps taken to normalize the raw data - can impact the accuracy of the network inference algorithms here considered. Full details of the normalization procedures applied are given in the Material and methods of he paper.

**Figure 14:**
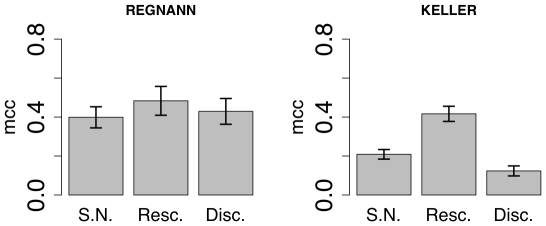
Accuracy (MCC) scores of RegnANN (left) and KELLER (right) on synthetic Erdös-Rényi networks with 100 nodes, SLC data generation and data ratio equal to 150, while varying data normalization.

Figure 14 shows the accuracy (MCC) of RegnANN and KELLER while varying the data normalization applied to the synthetic levels. The performance of KELLER significantly depends on the data normalization applied. In the case of data discretization, KELLER scores 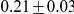, while an MCC value of 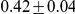 is recorded in the case of linear rescaling. Finally, if statistical normalization is applied to the data, KELLER scores 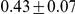. On the contrary, the accuracy of RegnANN is invariant (taking into account the error of the measure) with regards to the data normalization schema applied.

### Effect of variable binarization threshold

In this section we compare the performance of ARACNE, CLR and RegnANN varying the threshold applied to discretize the inferred adjacency matrix in terms of the the AUC (area under the curve) value.

**Figure 15:**
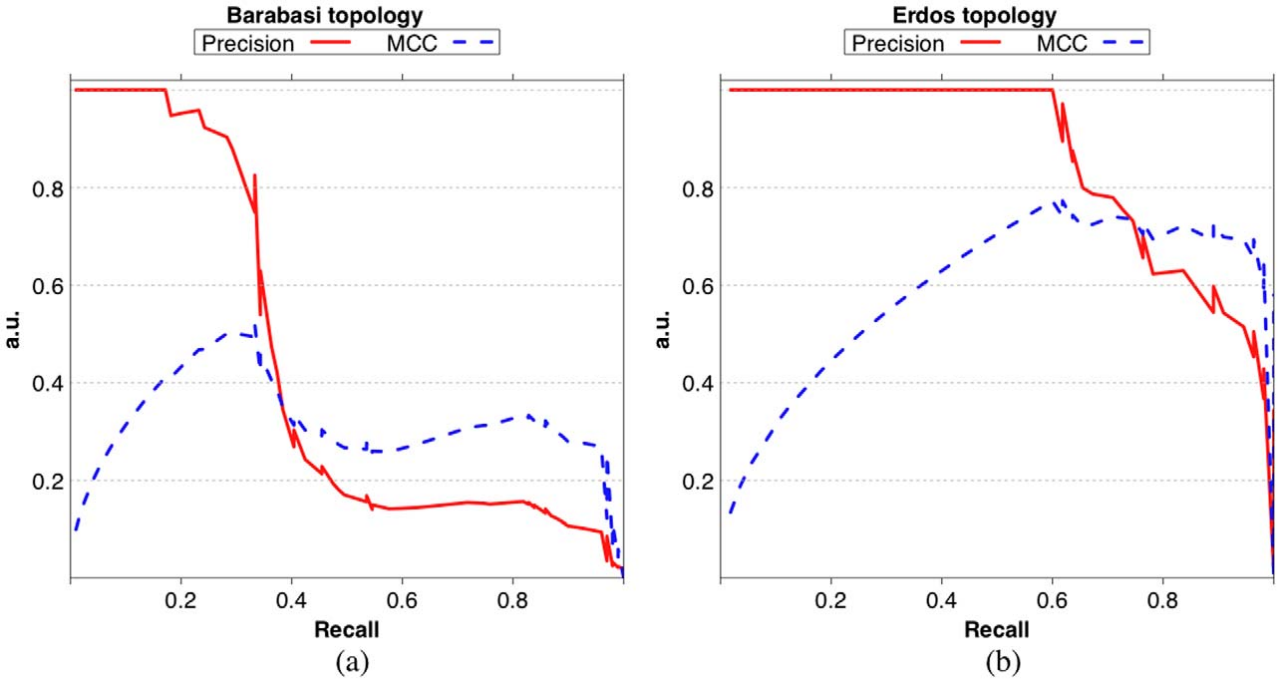
Precision-Recall curve (continuous line) and MCC-Recall curve (dashed line) for RegnANN on a synthetic Barabasi network (a) and on a synthetic Erdös-Rényi network (b), 100 nodes, 100% data ratio and SLC data generation.

Figure 15 shows a sample Precision-Recall curve (continuous line) and MCC-Recall curve (dashed line) for RegnANN on a synthetic Barabasi network (left) and on a synthetic Erdös-Rényi network (right). In the given example we considered 100 nodes, 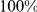 data ratio and SLC data generation. The AUC values for in the case of Barabasi network are: 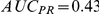 and 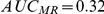. For the given Erdös-Rényi network the AUC values are: 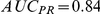 and 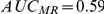. The figure shows curves that are in substantial agreement with the results reported in the previous section: the accuracy of the inference task strongly depends on the topology of the network.

**Figure 16:**
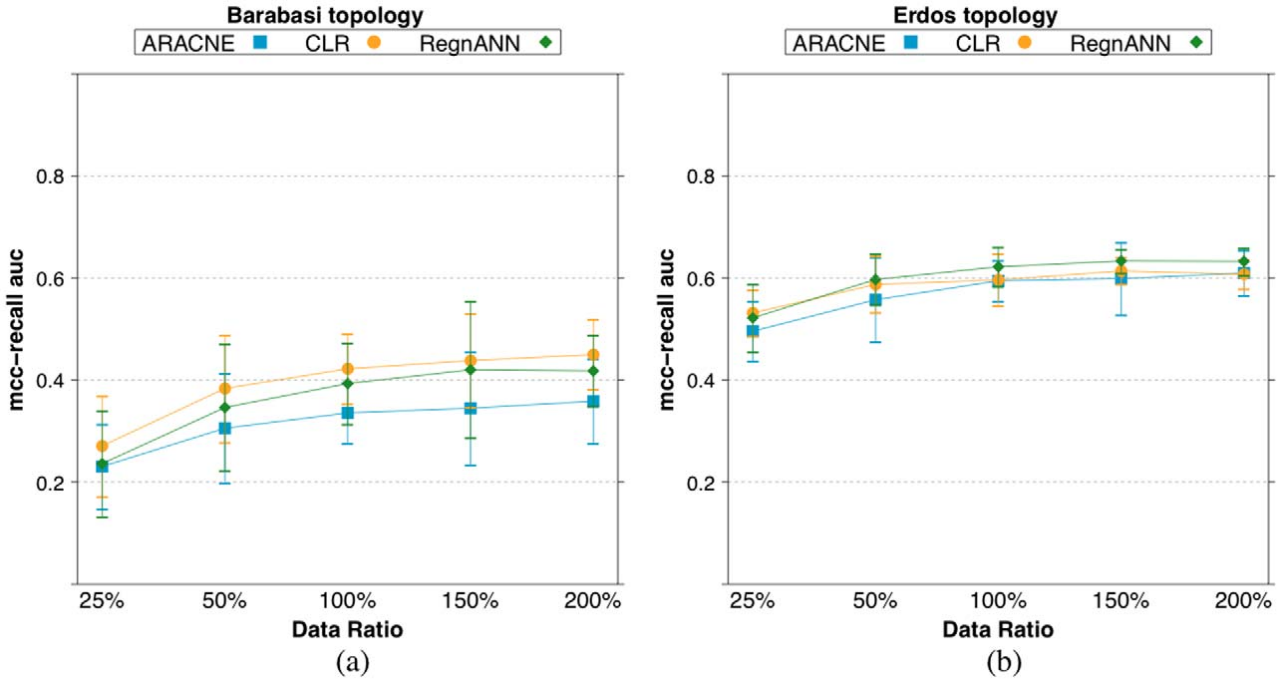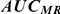 scores for ARACNE, CLR and RegnANN on a synthetic Barabasi network (a) and on a synthetic Erdös-Rényi network (b), 100 nodes and SLC data generation, varying data ratio.

Figure 16 shows the mean 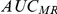 scores for ARACNE, CLR and RegnANN on a synthetic Barabasi network and on a synthetic Erdös-Rényi network, 100 nodes and SLC data generation, varying data ratio.

The figure indicates that the mean performance (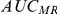) of the three methods obtained varying the discretization threshold are equivalent considering the confidence intervals, e.g.: in the case of data ratio 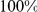 and Barabasi networks ARACNE scores 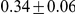, CLR scores 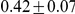 and RegnANN scores 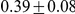. In the case of Erdös-Rényi networks, data ratio 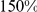 ARACNE scores 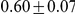, CLR scores 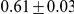 and RegnANN scores 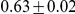.

**Figure 17:**
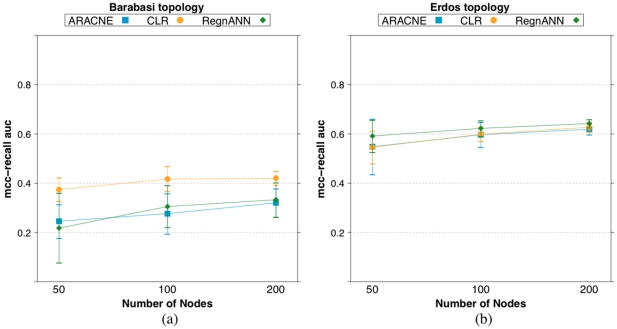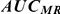 scores for ARACNE, CLR and RegnANN on synthetic networks varying the number of nodes, while keeping constant the data ratio (100%). (a) Synthetic Barabasi topology, GES data generation. (b) Synthetic Erdös-Rényi network, SLC data generation.

Figure 17 summarizes the 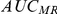 scores obtained by the three inference algorithms varying the number of nodes in the synthetic network. In the case of Barabasi networks, GES data generation, CLR tends of perform better than the other two methods. However, this phenomenon may not be strictly statistically significant, e.g.: with 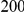 nodes, ARACNE scores 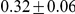, CLR scores 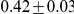, while RegnANN scores 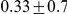. In the case of Erdös-Rényi networks, the three methods are equivalent, e.g.: considering 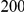 nodes, ARACNE scores 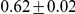, CLR scores 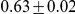 while RegnANN scores 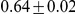.

**Figure 18:**
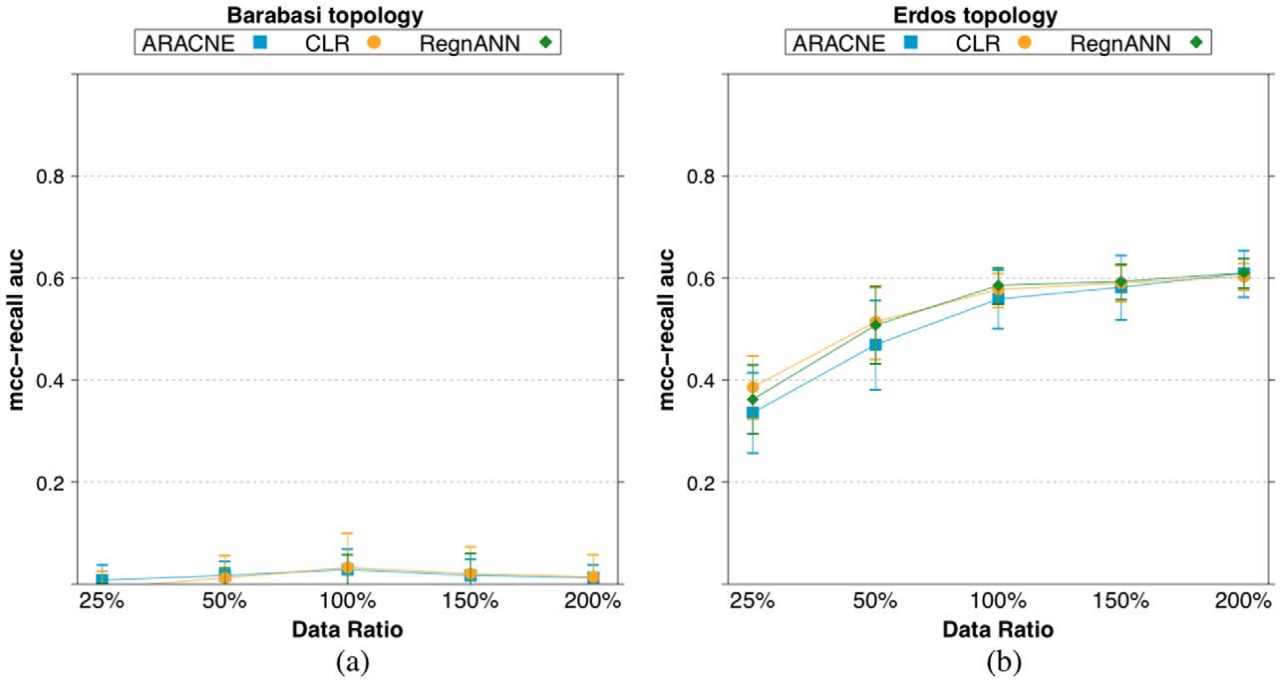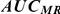 scores for ARACNE, CLR and RegnANN on a synthetic Barabasi network with power-law coefficient equal to 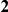 (a) and on a synthetic Erdös-Rényi network (b) with mean degree equal to 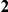. Here we consider 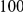 nodes and SLC data generation, while we vary the data ratio.

Figure 18 shows the mean 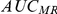 scores for ARACNE, CLR and RegnANN on a synthetic Barabasi network generated using a power-law exponent equal to 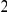 and on a synthetic Erdös-Rényi network with mean degree equal to 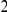. We consider 100 nodes and SLC data generation, while we vary the data ratio. In agreement with the results shown in Section Mean Degree and Power-law Coefficient, the figure indicates that increasing the power-law coefficient for the synthetic Barabasi network is detrimental to the performance of all the inference algorithm tested: ARACNE, CLR and RegnANN show mean 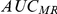 scores of 0, random chance. On the other hand, the three methods are less affected by increasing the mean degree of the synthetic Erdös-Rényi network, as also noticed previously.

### *Escherichia coli* transcriptional subnetworks

Table 4 summarizes the results obtained on a selection of *Escherichia coli* gene subnetworks [32] for the four inference algorithms. In absence of a gold standard data (not available in most realistic biological applications) for the estimation of the threshold for the binarization of the adjacency matrix, we arbitrarily set such threshold to 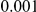 for ARACNE and CLR. This is done in the hypothesis that a value different from zero indicates meaningful interaction. It is important to stress the fact that the direction of the interaction is discarded as only symmetric matrices are considered. In the case of RegnANN, we set this threshold to 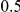 (we hypothesize that correlation values bigger than 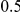 indicate meaningful interaction). In Section Selecting the Binarization Threshold we briefly discuss a possible strategy to infer the optimal threshold and the related shortcomings.

**Table 4:** MCC scores of the different network inference algorithms on the selected *Escherichia coli* network modules.

ID	Density	N.N.	N.L.	ARACNE	CLR
81	0.245	7	12	0.78	0.45
6	0.189	13	32	0.13	0.29
12	0.180	10	18	0.43	0.42
75	0.133	16	34	0.10	0.24
88	0.100	19	36	0.00	0.17
96	0.001	104	18	0.08	0.02
94	0.000	81	2	0.09	0.02

ID	Density	N.N.	N.L.	KELLER	RegnANN (Err)
81	0.245	7	12	−0.12	0.4 (0.1)
6	0.189	13	32	0.02	0.3 (0.1)
12	0.180	10	18	0.63	0.32 (0.00)
75	0.133	16	34	0.10	0.23 (0.08)
88	0.100	19	36	−0.07	−0.05 (0.02)
96	0.001	104	18	0.00	0.00 (0.01)
94	0.000	81	2	0.15	0.026 (0.001)

Column ID indicates the id of the network module as in [32], column Density the density of the module is the ratio of the number of links to the square of the number of nodes. Column N.N. indicates the nuber of nodes in the subgraph, while N.L. the number of links.

While ARACNE, CLR and KELLER are deterministic algorithms - given a particular input, the algorithm will always produce the same output, always passing through the same sequence of states - RegnANN may produce different results depending on the random initialization of the weights in the ensemble of multi-layer perceptrons. Thus, in order to smooth out possible local minima, we adopted a majority voting schema: for each network module, the RegnANN algorithm is applied 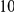 times and the inferred adjacency matrices accumulated. The final topology is obtained by selecting those links that appeares with a frequency higher than 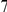. The entire procedure is repeated 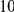 times, the final prediction is estimated as the mean and the associated error as twice the standard deviation of the 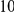 independent runs. Gene expression values are linearly rescaled in 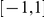.

Table 4 indicates great variability of the MCC scores across the different network modules for all the inference methods tested. ARACNE scores range from 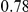 (module 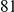) to 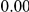 (module 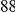). CLR values range between 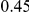 and 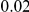 for module 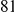 and 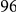 respectively. KELLER scores range between 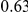 and 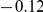 (module 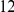 and module 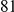 respectively). Finally RegnANN scores range between 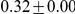 (module 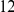, in this case the error associated to the measure is 0: the very same result is obtained for all repetitions) and 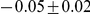 (module 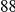). It is interesting to note that the MCC score varies unevenly for the reference inference algorithms with respect to the module network density (the ratio of the number of links to the square of the number of nodes), e.g.: ARACNE scores 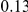 on module 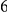 (density 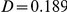) and scores 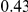 on module 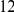 (density 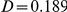). On the same two modules, CLR scores 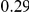 and 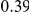 respectively while KELLER scores 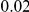 and 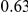. On the other hand, RegnANN scores are more homogeneous: they read 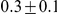 and 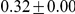 on module 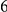 and module 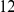 respectively. These results suggest that the correctness of the inferred network depends on the topological properties of the modules (the very same expression values are used to infer the different gene sub-networks), in accordance to findings in [4].

### Selecting the binarization Threshold

In this section we analyze the problem of optimal threshold selection for the binarization of the inferred adjacency matrix. Generally, the threshold cutoff value should be estimated for each data-set (network) or it should be derived from a gold standard data, which is not available in most realistic biological applications. An obvious solution to this problem is to adopt a training/validation schema: ground-truth data is used to infer the optimal threshold value while external data is used to verify the reconstruction accuracy. Here, for each module in Table 4 and for three inference algorithms (ARACNE, CLR and RegnANN), we partition the *Escherichia coli* data-set in training data (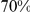) and validation data (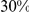). The training data is used to infer the best threshold cutoff value: we perform a grid search for threshold values in 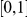 and we pick the value granting the best reconstruction score. The validation set is used to verify the accuracy (MCC) of the inference algorithms. This procedure is repeated 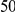 times randomly partioning the *Escherichia coli* data-set each time. Scores are expressed as the mean value, while the error as twice the standard deviation.

Table 5 shows the optimal threshold cutoff values for the three inference methods (ARACNE, CLR and RegnANN) and for the *Escherichia coli* submodules as in Table 4.

**Table 5:** Mean values of the optimal threshold cutoff scores for the three inference methods (ARACNE, CLR and RegnANN) varying the *Escherichia coli* submodules.

ID	ARACNE	Err.	CLR	Err.	RegnANN	Err.
81	0.04	0.01	0.61	0.07	0.01	0.01
6	0.35	0.06	0.15	0.01	0.22	0.03
12	0.12	0.01	0.4	0.2	0.36	0.01
75	0.42	0.02	0.5	0.2	0.59	0.03
88	0.39	0.01	0.33	0.06	0.43	0.03
96	0.87	0.01	0.74	0.01	0.66	0.01
94	0.81	0.01	0.62	0.05	0.95	0.01

The table indicates that the optimal threshold value depends on the algorithm adopted and the submodule considered.

Table 6 shows the accuracy (MCC) obtained on the training set and on the validation set with the optimal threshold cutoff value. Scores are obtained varying inference method and *Escherichia coli* submodule. Table 6 shows values that vary considerably depending on the subnetwork selected and the inference algorithm adopted. It is interesting to note that although the variance of the MCC scores is small (the column Error in the table) the accuracy on the validation set is often higher than the corresponding the training set accuracy score, e.g.: module 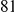, 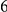, 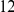, 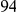 in the case of ARACNE; module 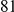 and module 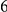 in the case of CLR; module 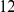 in the case of RegnANN. On the other hand, there are cases where good training accuracy scores are not matched with comparable validation accuracies, e.g.: module 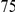, 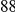, 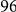 in the case of ARACNE; module 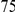, 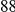 in the case of CLR. This results indicates both a good stability in replicating the results using the estimated optimal threshold (small error), and a tendency to both poorly fit and over fit the data.

**Table 6:** Mean accuracy (MCC) obtained on the training set and on the validation set for the different inference methods and network submodules.

ARACNE				
ID	Training	Error	Validation	Error
81	0.21	0.01	0.57	0.03
6	0.04	0.01	0.19	0.02
12	0.26	0.01	0.32	0.03
75	0.54	0.02	0.14	0.01
88	0.75	0.01	0.03	0.01
96	0.71	0.01	0.27	0.02
94	0.05	0.01	0.40	0.10

	CLR			
ID	Training	Error	Validation	Error
81	0.16	0.01	0.57	0.02
6	0.03	0.01	0.31	0.01
12	0.36	0.01	0.36	0.01
75	0.46	0.01	0.13	0.01
88	0.71	0.01	0.08	0.01
96	0.03	0.01	0.04	0.01
94	0.11	0.01	0.03	0.01

	RegnANN			
ID	Training	Error	Validation	Error
81	0.24	0.01	0.23	0.01
6	0.34	0.01	0.10	0.01
12	0.22	0.01	0.31	0.02
75	0.40	0.01	0.17	0.01
88	0.23	0.01	0.08	0.01
96	0.03	0.01	0.02	0.01
94	0.12	0.01	0.00	0.01

Although outside the scope of this work, this preliminary evaluation indicates that in the case of biological data, learning the optimal threshold value via standard machine leaning methods is not straightforward: presence of noise in the data and the high complexity of the domain often cause selection bias. This is the key point that lead us focus on estimating the structures of the interaction between genes rather than the detailed strength of these interactions.

## Discussion

In this work we presented a novel method for network inference based on an ensemble of multi-layer perceptrons configured as multi-variable regressor (RegnANN). We compared its performance to the performance of three different network inference algorithms (ARACNE, CLR and KELLER) on the task of reverse engineering the gene network topology, in terms of the associated MCC score. The proposed method makes no assumptions about the nature of the relationships between the variables, capturing high-order dependencies between expression patterns and the direction of the interaction, as shown on selected synthetic toy examples. Our extensive evaluation indicates that the newly introduced RegnANN shows accuracy and stability scores that compare very favorably with all the other inference methods tested, often outperforming the reference algorithm in the case of fixed binarization threshold. On the other hand, considering all the possible thresholds for the binarization of the inferred adjacenci matrix (the AUC score) the differences among the tested methods tend to become irrelevant. Our evaluation on synthetic data demonstrates that various factors influence the performance of the inference algorithms adopted: the topology of network, its size and its complexity, the amount of data available, the normalization procedure adopted. Generally, these are only a few of the factors that may influence the outcome of a network inference algorithm; they may not be limited to the relative small set of parameters explored here.

Results on the biological data confirm that the correctness of the inferred network depends on the topological properties of the modules: very different accuracy results are obtained on the different submodules of *Escherichia coli*, although the very same expression values are used to infer the different gene sub-networks. Our experiments indicate great variability of the scores of the reference inference algorithms across the different *Escherichia coli* sub-modules. On the other hand, RegnANN scores are more homogeneous, decreasing as the density of the module decreases.

Finally, we tested the possibility of applying standard machine leaning methods to learn the optimal binarization threshold value. Our preliminary evaluation indicates that this is not a straightforward task: presence of noise in the data and the high complexity of the biological domain often cause selection bias.

The robustness of RegnANN performance recorded across the board and the agreement between results obtained with this new methods on both synthetic and biological data are promising and they stimulate its application to a wider range of problems.

## Materials and Methods

### RegnANN: Network Inference Using ANN

**Figure 19:**
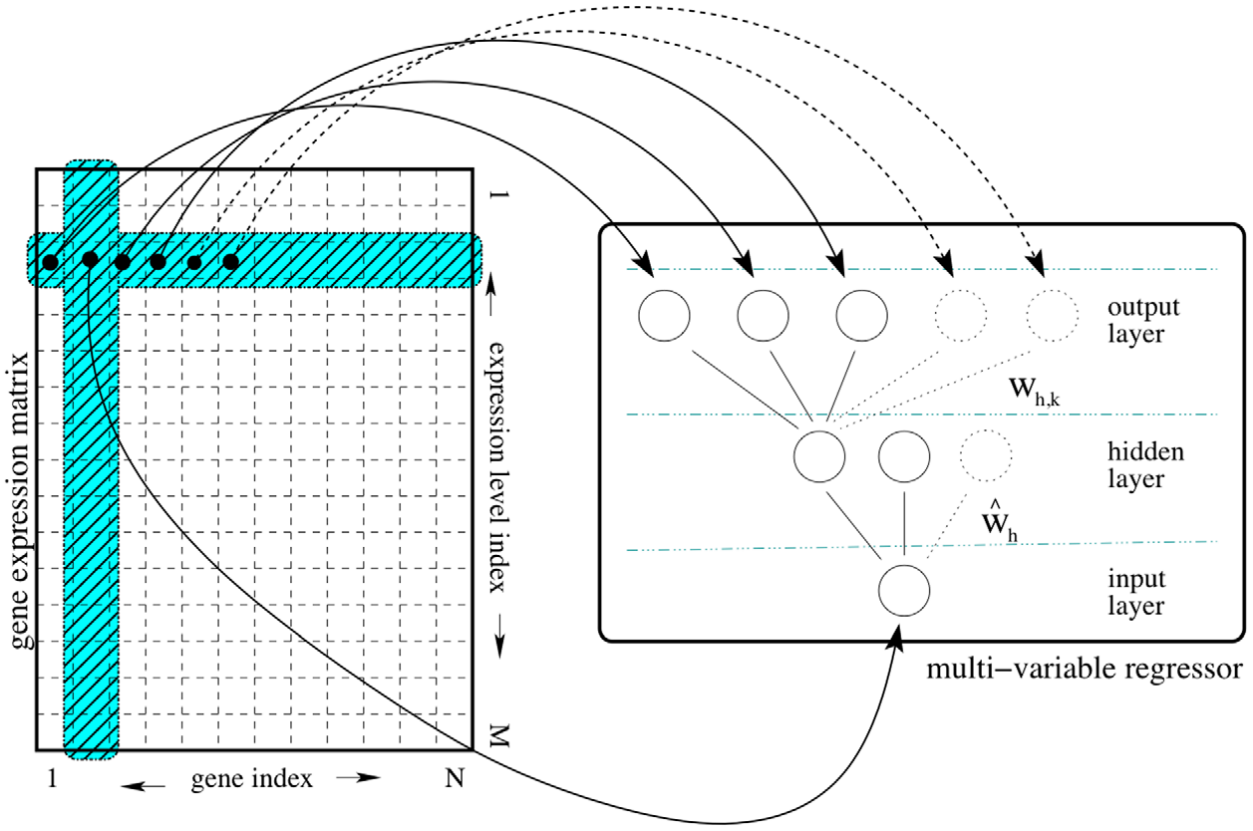
The *ad hoc* procedure proposed to build the training input/output patterns starting from a gene expression matrix. Each input pattern corresponds to the expression value for the selected gene of interest. The corresponding output pattern is the vector of expression values for all the other genes for the given row in the gene expression matrix. The right part of the figure schematizes the multi-variable regressor: a feed-forwardad multilayer perceptron with 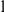 input neuron, 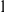 layer of 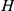 hidden units and 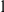 layer of 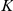 output units; 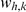 are the weights associated with the links between the output layers and the hidden layer and 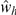 the weights of the links between the input neuron and the hidden layer.

To infer gene regulatory networks we adopt an ensemble of feed-forward multilayer perceptrons. Each member of the ensemble is essentially a multi-variable regressor (one to many) trained using an input expression matrix to learn the relationships (correlations) among a target gene and all the other genes. Formally, let us consider the multilayer perceptron as in Figure 19 (right): 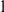 input neuron 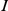, 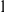 layer of 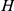 hidden units and 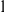 layer of 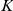 output units. Indicating with 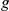 the activation function of each unit and 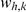 the weights associated with the links between the output layers and the hidden layer and with 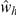 the weights of the links between input neuron and hidden layer, the value 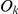 for the output unit 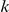 can be calculated as follows:
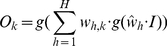
(1)The value 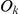 is the inferred interaction between the corresponding gene 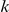 and the gene associated with the input neuron 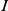. We proceed in determining the interactions among genes separately and then we join the information to form the overall gene network. From each row of the gene expression matrix we build a set of input and output patterns used to train with back-propagation [33] a selected multilayer perceptron. Each input pattern corresponds to the expression value for the selected gene of interest. In this work we consider gene expression matrices of dimension 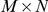, i.e. 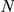 genes whose expression levels are recorded 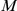 times; expression levels are normalized in the interval 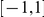. The output pattern is the row-vector of expression values for all the other genes for the given row in the gene expression matrix (Figure 19). By cycling through all the rows in the matrix, each regressor in the ensemble is trained to learn the correlations among one gene and all the others. Repeating the same procedure for all the columns in the expression matrix, the ensemble of multi-variable regressors is trained to learn the correlations among all the genes. The procedure of learning separately the interactions among genes is very similar to the one presented in [20], where the authors propose to estimate the *neighborhood* of each gene (the correlations among one gene and all the others) independently and then joining these neighborhoods to form the overall network, thus reducing the problem to *a set of identical atomic optimizations*.

We build 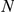 (one for each of the 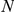 genes in the network) multilayer perceptrons with one input node, one layer of hidden nodes and one layer of 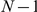 output nodes, adopting the hyperbolic tangent as activation function. The input node takes the expression value of the selected gene rescaled in 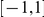. The number of hidden nodes is set to the square root of the number of inputs by the number of outputs. This value is to be considered a rule of thumb granting enough hidden units to solve the regression problem and allowing dynamical adaptation of the structure of RegnANN to the size of the biological network under study. The output layer provides continuous output values in the range 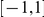.

The algorithm of choice for training each multi-layer perceptron is the back-propagation algorithm [33]. The back-propagation is a standard algorithm for learning feed-forward multilayer perceptrons that essentially looks for the minimum of the error function in the weight space using the method of gradient descent. The error function is defined as the difference between the output of each neuron in the multilayer perceptron and its expected value. The back-propagation algorithm starts with the forward-propagation of the input value in the multilayer perceptron, followed by the backward propagation of the errors from the output layer toward the input neuron. The algorithm corrects the weight values according to the amount of error each unit is responsible for. Formally, the weight values at learning epoch 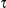 are updated as follows:
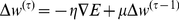
(2)To keep the notation simple 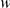 refers to both the weights associated with the links between the output layers and the hidden layer and with the weights of the links between input neuron and hidden layer. 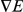 refers to the gradient of the error in weight space. 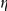 is the learning rate; 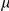 is the momentum.

Although back-propagation is essentially a heuristic optimization method and alternatives such as Bayesian neural network learning [34] have more sound theoretical basis, in the proposed multi-variable regression schema the simple back-propagation algorithm allows us to design a far less complex system. This is due to how Bayesian neural network learning handles the regression problem. As indicated in [35]: “Networks are normally used to define models for the conditional distribution of a set of target values given a set of input values.[…]. For regression and logistic regression models, the number of target values is equal to the number of network outputs.” This implies that in the case of Bayesian learning an extra procedure is required to discretize the target values from the continuous range 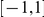 and that for each ensemble member the layer of output neurons (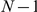 in the case of back-propagation) has to be translated into a matrix of neurons of size 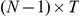, where 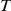 is the number of desired target values. Accordingly, also the hidden layer becomes a matrix of neurons, each one with its own set of parameters. Thus, in the context of multivariable regression, adopting back-propagation allow us to design a lower complexity inference system limiting issues related to high dimensional settings.

The learning parameters we use to train each multi-layer perceptron are as follows: learning rate equal to 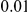; momentum equal to 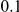, learning epochs equal to 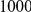. These values are evaluated empirically during preliminary tests on synthetic data. In section 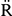
egnANN: varying learning parameters
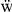
e show how the performance of the proposed method depends on the choice of the learning parameters.

Once the ensemble is trained, the topology of the gene regulatory network is obtained by applying a second procedure. Considering each gene in the network separately, we pass a value of 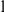 to the input neuron of the correspondent multilayer perceptron, consequently recording its output values. The continuous output values in the range 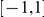 represent the expected normalized expression values for the other genes (its neighborhood). This procedure basically aims at verifying the correlation between the input gene and all the others: assuming the input gene maximally expressed (the value 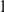), an output value of (i.e.) 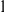 indicates that the correspondent gene will be also maximally expressed, thus indicating perfect correlation between the two genes. An output value of (i.e.) 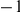 indicates that the correspondent gene will be maximally under-expressed: perfect anti-correlation of the two genes. Thus, the continuous output values in the range 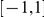 are interpretable in terms of positive correlation (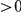), anti-correlation (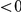) and no-correlation (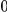). By cycling this procedure through all the ensemble members in the regression system, we obtain 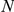 (one for each of the 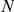 genes in the network) vectors of length 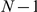 of continuos values in 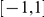. The correlation matrix is obtained by correctly joining the N vectors. It is important to note that all the values of the diagonal of the adjacency matrix are equal to 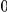 by construction: this procedure does not allow discovering of gene self correlation (regulation) patterns, but only correlation patterns among different genes. Finally the adjacency matrix of the sought gene network is obtained by thresholding the correlation coefficients.

To improve the general efficiency of the algorithm and thus to allow a systematic comparison of its performance with the other gene network reverse engineering methods tested, we implemented the ANN based regression system using the GPGPU programming paradigm. A reference implementation of the RegnANN algorithm is available at http://sourceforge.net/projects/regnann/files/. The source code is distributed according to the GPLv3 license (open-source).

### Alternative inference methods

As reference methods we select three alternative algorithms widely used in literature: ARACNE, CLR and KELLER.

*KELLER* is a kernel-reweighted logistic regression method [20] introduced for reverse engineering the dynamic interactions among genes based on the time series of their expression values. It estimates the neighborhood of each gene separately and then it joins the neighborhoods to form the overall network. The approach aims at reducing the network inference problem to a set of identical atomic optimizations. KELLER makes use of the 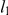-regularized logistic regression algorithm and it operates modeling the distribution of interactions between genes as a binary pair-wise Markov Random Field.

With respect to other inference methodologies, KELLER adopt a fixed threshold to discretize the inferred adjacency matrix while it performs an optimization of the regularization weight lambda controlling the sparsity of the solution by maximizing a Bayesian Information Criterion (BIC). The authors apply a grid search on a selection of possible parameter values. In our work, we adopt the very same procedure: we use the same fixed discretization threshold for the binarization of the adjacency matrix, while we select the optimal solution by maximizing the BIC via a grid search for the optimal value of lambda (the very same value range used in [20]). This is done for each inference task.

As indicated in [20], the KELLER algorithm approximates the dynamic rewiring of the gene networks topology by borrowing “information across time by reweighting the observations from different time points and then treating them as if they were i.i.d. observations. Intuitively, the weighting should place more emphasis on observations at or near time point t with weights becoming smaller as the observations move further away from time point t. ” Thus, this procedure relies in fact on a sequence of static topologies that do not differ greatly one another (one of the KELLER algorithmic assumptions is that the time-evolving network varies smoothly across time). In this work we consider only one fixed (static) topology for each inference task subsequently measuring the correctness of the result.

*ARACNE* is a general method able to address a wide range of network deconvolution problems - from transcriptional [8] to metabolic networks [10] - that was originally designed to scale up to the complexity of regulatory networks in mammalian cells. The method makes use of an information theoretic approach to eliminate the majority of indirect interactions inferred by co-expression methods. ARACNE removes the vast majority of indirect candidate interactions by applying a well-known information theoretic property: the data processing inequality [36].

Here we use the reference implementation of the algorithm provided in [37] with default value for the data processing inequality tolerance parameter. In Section “ARACNE: varying learning parameters” we explore the influence of the tolerance parameter on a sample testbed.

As many other methods, ARACNE relies on the definition of a threshold for the binarization of the adjacency matrix. In absence of a good heuristic for defining such threshold, on the synthetic data-sets we will adopt the area under the curve (AUC) as performance metric.

*CLR* is an extension of the relevance networks class of algorithms [9], which predicts regulations between transcription factors and genes by applying the mutual information score. CLR proposes an adaptive background correction step that is added to the estimation of mutual information. For each gene, the statistical likelihood of the mutual information score is computed within its network context. Then, for each transcription factor-target gene pair, the mutual information score is compared to the context likelihood of both the transcription factor and the target gene, and turned into a z-score. We adopt the reference implementation of the algorithm provided in [37]. As in the case of ARACNE, in absence of a good heuristic for defining a binarization threshold for the inference of the adjacency matrix, on the synthetic data-sets we will adopt the area under the curve (AUC) as performance metric.

### Performance Metric

When the performance of a network inference method is evaluated, it is common practice to adopt two metrics: precision and recall. Recall indicates the fraction of true interactions correctly inferred by the algorithm, and it is estimated according to the following equation:
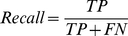
(3)where TP indicates the fraction of *true positives*, while FN indicates the fraction of *false negatives*.

On the other hand, precision measures the fraction of true interactions among all inferred ones, and it is computed as:
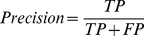
(4)where FP indicates the ratio of *false positives*.

In this work we adopt the Matthews Correlation Coefficients - MCC [27], [28]: this is a measure that takes into account both true/false positives and true/false negatives and it is generally regarded to as a balanced measure, useful specially in the case of unbalanced classes (i.e.: different number of positive and negative examples).

The MCC is in essence a correlation coefficient between the observed and predicted binary classifications: it returns a value between 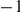 and 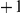. A coefficient value equal to 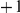 represents a perfect prediction, 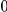 indicates an average random prediction while 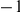 an inverse prediction [27], [38]. In the context of network topology inference the observed class is the true network adjacency matrix, while the predicted class is the inferred one.

The Matthews Correlation Coefficient is calculated as:
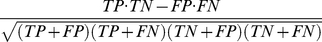
(5)Recently MCC has also been used for comparing network topologies [28], [39].

### Data

We benchmark the reverse engineering algorithms here considered using both synthetic and biological data.

### Synthetic Data

**Figure 20:**
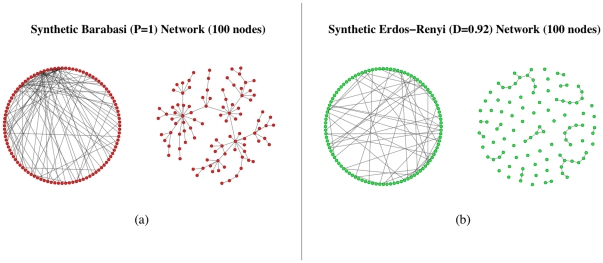
Sample network topologies: (a) Barabasi Network with 100 nodes (power-law exponent 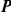 equal to 1); (b) Erdös-Rényi network, 100 nodes and average degree (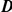) egual to 0.92.

The synthetic data sets are obtained starting from an adjacency matrix describing the desired topology of the network. Here we consider two different network topologies: Barabasi-Albert [29] and Erdös-Rényi [30]. Network graphs are generated using the *igraph* extension package to the GNU R project for Statistical Computing [40]. Figure 20 shows two sample network topologies. In this work we focus our analysis on undirected and unweighted graphs: we are interested in estimating the structures of interaction between nodes/genes, rather than the detailed strength or the direction of these interactions. Thus, we consider only symmetric and discrete adjacency matrices, representing with a value of 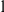 the presence of a link between two nodes. A value equal to 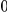 in the adjacency matrix indicates no interaction.

Once the topology of the network is (randomly) generated, the output profiles of each node are generated according to two different approaches: the first one considers only linear correlation among selected genes (SLC), the second one is based on a gene network/expression simulator recently proposed to assess reverse engineering algorithms (GES [41]). In order to account for the intrinsic underdetermination of the task of network inference, we focus on synthetic data mimicking plausible submodules of larger networks: relatively small networks with a number of nodes ranging between 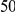 and 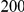 and a number of expression profiles ranging from 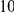 to 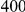. Thus, we settle our analysis in a scenario of reduced search space/extended amount of independent information.

*Simple Linear Correlation (SLC):* similarly to the simulation of gene expression data presented in the supplementary material of [42], we consider a set of *seed* expressions (a matrix 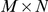 - 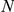 genes which expression profiles are recoded 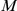 times - with values uniformly distributed in 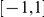) and the desired topology expressed by the adjacency matrix 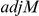 (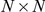). The matrix 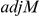 contains only zeros and ones: a value of one indicates a link between the corresponding genes. The gene expression profiles (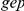, a matrix 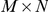) are calculated as:
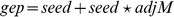
(6)where the symbol ‘
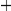
’ indicates element-wise summation and the symbol ‘
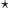
’ indicates row-column matrix multiplication. With this method, the *seed* expression columns are linearly correlated (correlation equal to 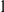) with the columns of the same matrix as described by the discrete input adjacency matrix *adjM*.

*Gene Expression Simulator (GES):* this second methodology is based on a gene network simulator recently proposed to assess reverse engineering algorithms [41]. Given an input adjacency matrix, the network simulator uses fuzzy logic to represent interactions among the regulators of each gene and it adopts differential equations to generate continuous data. As in [8], we obtain synthetic expression values of each gene 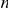 (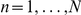) by simulating its dynamics until the expression value reaches its steady state. We obtain 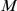 different values for each gene by repeating the process 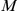 times and recording the expression value at steady state. The synthesis of each gene profile is randomly initialized by the simulator.

### *Escherichia coli* Transcriptional Network

The task for the biological experiments is the inference of a few transcriptional subnetworks of the model organism *Escherichia coli* starting from a set of steady state gene expression data. The data are obtained from different sources and they consist of three different elements, namely the whole *Escherichia coli* transcriptional network, the set of the transcriptional subnetworks and the gene expression profiles to infer the subnetworks from. The *Escherichia coli* transcriptional network is extracted from the RegulonDB (http://regulondb.ccg.unam.mx/) database, version 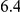 (2010) and it consists of 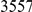 experimentally confirmed regulations between 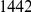 genes, amongst which 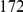 transcription factors. The 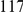 subnetworks are defined in [43]: in our experiments we use 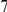 of these subnetworks, with a number of genes ranging from 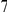 to 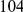. Information about number of genes and number of links for each subnetwork is reported in Table 4, Section Results. The expression data have been originally used in [9] and they consist of 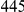 *Escherichia coli* Affymetrix Antisense2 microarray expression profiles for 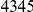 genes, collected under different experimental conditions such as pH changes, growth phases, antibiotics, heat shock, varying oxygen concentrations and numerous genetic perturbations. MAS5 preprocessing is chosen among the available options (MAS5, RMA, gcRMA, DChip).

### Data Discretization

A number of sources of noise can be introduced into the microarray measurements, e.g. during the stage of hybridization, digitization and normalization. Therefore, it is often preferred to consider only the qualitative level of gene expression rather than its actual value [20]: gene expression is modeled as either being up-regulated (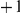) or down-regulated (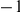) by comparing the given value to a threshold. For example, in [44] it is shown that binarizing gene expression data leads to classification outcomes very similar to the results obtained on real-valued data.

In this work we compute the discrete value of the expression for each of the 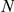 genes at each of the 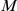 steps as the sign of the difference of the expression values of the given gene at step 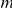 and step 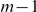.

### Data Rescaling

Generally, when a scaling method is applied to the data, it is assumed that different sets of intensities differ by a constant global factor [31]. It may also happen that the rescaling is a necessary step due to the inference method adopted, as in the case of SVM (Support Vector Machine) or ANN (Artificial Neural Network) classification/regression.

In this work we test two different data rescaling methods:

- *linear rescaling*: each gene expression column-vector is linearly rescaled between 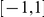;
- *statistical normalization*: each gene expression column-vector is rescaled such that its mean value is equal to 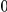 and the standard deviation equal to 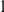.

We consider gene expression matrices of dimension 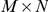: 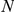 genes whose expression levels are recorded 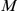 times.

### Experimenting with learning parameters

#### RegnANN: varying learning parameters

In this section we show how the tuning parameters of RegnANN impact its performance on a selected testbed: Barabasi networks with 100 nodes and SLC data generation. Accuracy scores (MCC) are calculated as mean of 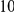 iterations. Error bars are omitted for clarity. Gene expression profiles are rescaled linearly in 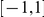.

**Figure 21:**
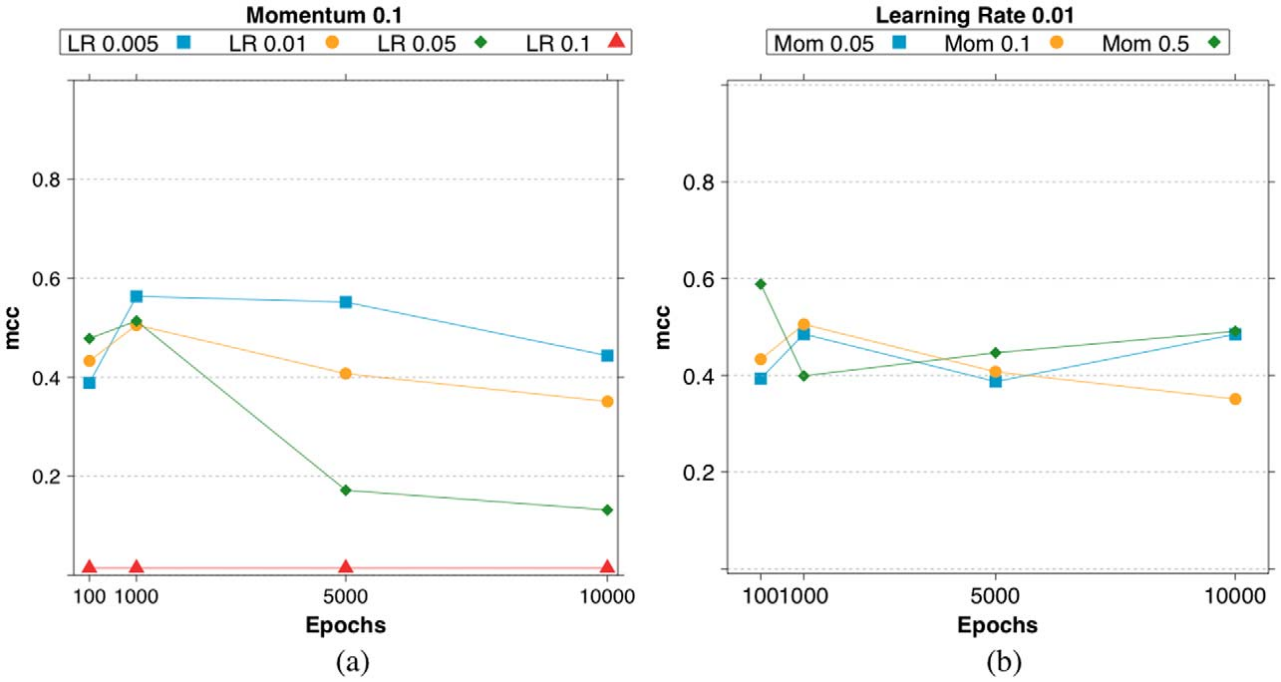
Accuracy (MCC) scores for RegnANN on synthetic Barabasi networks with 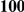 nodes and SLC data generation. (a) Fixed momentum (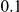), varying learning rate and training epochs. (b) Fixed learning rate (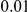), varying momentum and training epochs.

Figure 21 summarizes the MCC score of RegnANN obtained varying training epochs for fixed momentum and learning rate values.

**Figure 22:**
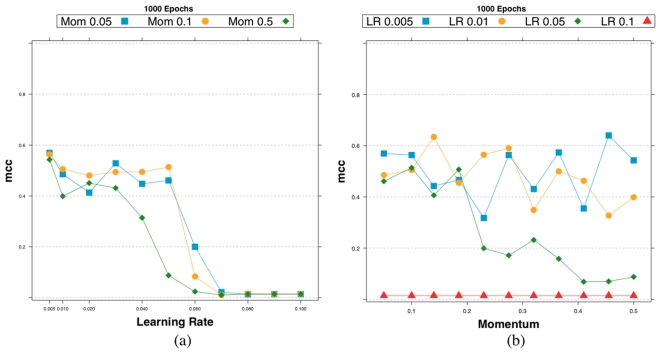
Accuracy (MCC) scores for RegnANN on synthetic Barabasi networks with 100 nodes, SLC data generation. We consider fixed training epochs (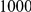) while varying (a) learning rate and (b) momentum.

Figure 22 summarizes the MCC score of RegnANN obtained varying momentum and learning rate for fixed training epochs value.

The two set of figures indicate that the values for the learning parameters adopted in the evaluation of the performance of RegnANN (momentum = 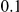, learning rate = 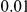, training epochs = 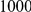) are chosen prudently.

#### ARACNE: varying learning parameters

In this section we explore the influence of the tolerance parameter (eps) on a sample testbed. The reference implementation of ARACNE provided by [37] as R package [40] sets its value to 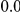.

We adopt the 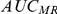 score (the AUC value for the MCC-Recall curve) as performance metric. 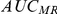 values are calculated as mean of 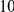 iterations. Error bars are calculated as twice the standard deviation.

**Figure 23:**
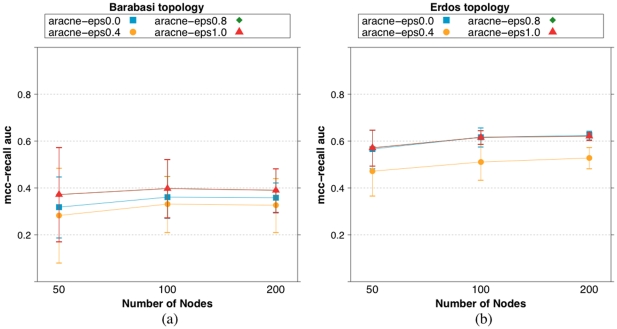
Mean 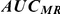 varying the eps value, the topology of the network and the number of nodes. Gene expression profiles are rescaled linearly in 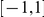. (a) Barabasi topology. (b) Erdös-Rényi networks.

Figure 23 shows the mean 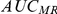 (the AUC value for the MCC-Recall curve) varying the eps value in 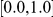, the topology of the network and the number of nodes. Gene expression profiles are rescaled linearly in 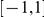. We consider fixed data ratio equal to 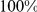.

The figure indicates that for Barabasi networks no statistically relevant difference in the performance of ARACNE is recoded varying the eps parameter, e.g.: considering network size 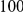 nodes, with eps value 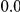 ARACNE scores 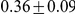; with eps value 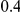 ARACNE scores 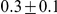; with eps value 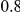 ARACNE scores 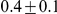; with eps value 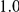 ARACNE scores 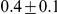. In the case of Erdös-Rényi, setting the eps parameter value to 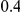 is detrimental for the mean accuracy of ARACNE, while all the other values tested result in equivalent performances, e.g.: considering network size 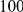 nodes, with eps value 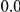 ARACNE scores 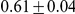; with eps value 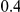 ARACNE scores 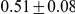; with eps value 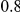 ARACNE scores 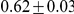; with eps value 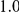 ARACNE scores 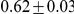.

The figure indicates that the value 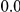 is a sound default for the eps parameter.
